# Single Vector System for Efficient N-myristoylation of Recombinant Proteins in *E. coli*

**DOI:** 10.1371/journal.pone.0010081

**Published:** 2010-04-09

**Authors:** Julian M. Glück, Silke Hoffmann, Bernd W. Koenig, Dieter Willbold

**Affiliations:** 1 Institute of Structural Biology and Biophysics, Research Centre Jülich, Jülich, Germany; 2 Institut für Physikalische Biologie, Heinrich-Heine-Universität Düsseldorf, Düsseldorf, Germany; University of California Davis, United States of America

## Abstract

**Background:**

N-myristoylation is a crucial covalent modification of numerous eukaryotic and viral proteins that is catalyzed by N-myristoyltransferase (NMT). Prokaryotes are lacking endogeneous NMT activity. Recombinant production of N-myristoylated proteins in *E. coli* cells can be achieved by coexpression of heterologous NMT with the target protein. In the past, dual plasmid systems were used for this purpose.

**Methodology/Principal Findings:**

Here we describe a single vector system for efficient coexpression of substrate and enzyme suitable for production of co- or posttranslationally modified proteins. The approach was validated using the HIV-1 Nef protein as an example. A simple and efficient protocol for production of highly pure and completely N-myristoylated Nef is presented. The yield is about 20 mg myristoylated Nef per liter growth medium.

**Conclusions/Significance:**

The single vector strategy allows diverse modifications of target proteins recombinantly coexpressed in *E. coli* with heterologous enzymes. The method is generally applicable and provides large amounts of quantitatively processed target protein that are sufficient for comprehensive biophysical and structural studies.

## Introduction

N-myristoylation is the covalent attachment of myristic acid to an NH_2_-terminal glycine residue of a protein substrate via an amide bond [1]. The reaction is catalyzed by N-myristoyltransferase (NMT), an essential enzyme of 50 to 60 kDa that is ubiquitously expressed in many organisms [2]. Following the discovery of N-myristoylation by Carr et al. [3] numerous N-myristoylated eukaryotic and viral proteins have been identified (reviewed in [1], [4]). Myristoylated proteins play key roles, for example in oncogenesis, cell signaling and infectivity of retroviruses.

Protein myristoylation has been linked to various biological functions. It is involved in membrane binding and targeting. Suppression of N-myristoylation can adversely affect the subcellular location of proteins. For example, myristoylated Gag precursor polyprotein of the spleen necrosis virus is anchored in the plasma membrane during proteolytic cleavage. In contrast, nonmyristoylated Gag mutants undergo aberrant processing resulting in failure of virus particle assembly and release [5]. N-myristoylation can have a strong impact on protein structure [6], e.g. it confers structural and thermal stability to the catalytic subunit of cAMP-dependent protein kinase [7], [8]. Thus, proteins that require such modification for biologic activity should be studied in their myristoylated form. Large amounts of functional protein, however, are often required for structural and biophysical studies. The most widely employed organism for recombinant protein production is *E. coli*. Often, this allows biosynthesis of diverse proteins at high yield and low cost. A major drawback of protein expression in bacterial hosts is the absence of eukaryote-specific co- and posttranslational modifications, e.g. due to lack of endogenous NMT activity.

Several alternatives are available for N-myristoylation of bacterially expressed proteins. Successful *in vitro* myristoylation on an analytical scale has been reported [9], [10]. Catalytic amounts of exogeneous NMT, protein substrate, and an excess of myristic acid are incubated during the reaction. Even though almost quantitative *in vitro* N-myristoylation of a 17 kDa protein has been observed after 8 hours of incubation, the reaction rate and efficiency appears to decrease with increasing size of the protein substrate [9]. Both the NMT and the target protein must be purified prior to the *in vitro* myristoylation and an additional purification step is necessary to remove the enzyme, unmodified protein and the fatty acid substrate after the reaction.

Higher yields of N-myristoylated proteins can be achieved by coexpression of target proteins with a heterologous N-myristoyltransferase in *E. coli* [11]–[17]. *In cell* N-myristoylation is triggered by supplementing the growth medium with the NMT substrate myristic acid. In the past, dual plasmid systems have been employed, where one plasmid carries an NMT gene while the other one codes for the target protein. Stable maintenance of both plasmids in the same cell, however, requires the use of two vectors with compatible replicons and different antibiotic resistance markers.

The ratio of codons present in the coding sequence of a heterologous gene product frequently proves inconsistent with the tRNA pool of *E. coli* (codon bias). Thus, the limited supply of certain tRNAs in *E. coli* often impedes efficient production of heterologous proteins especially from organisms with AT- and GC-rich genomes. Modified bacterial strains are available to overcome this problem. They contain an additional plasmid which encodes tRNAs that tend to get depleted during expression of the target gene. However, maintenance of the additional plasmid requires a distinct antibiotic resistance marker. Transformation of such an *E. coli* strain with two plasmids for expression of NMT and target protein, respectively, would therefore result in a system that subjects the host cell to three separate antibiotics. Exposure to multiple antibiotics further increases the level of stress on the bacterium and a reduced target protein yield is often observed. The situation may become even worse if isotope labeling of the target protein is desired necessitating bacterial growth and gene expression in minimal medium. The number of antibiotic resistance markers can be reduced either by optimizing codon selection in the gene of the target protein or by using a single vector with two expression cassettes in tandem for production of the NMT and the target protein. In the first case, the additional plasmid for production of rare tRNAs is no longer needed. Equally, the use of a bicistronic vector instead of two plasmids reduces the number of required antibiotic resistance markers by one.

Nef is an accessory protein of approximately 25 kDa encoded in the genome of human immunodeficiency virus 1 (HIV-1). It is a multifunctional protein that plays a crucial role in HIV pathogenesis and in disease progression to AIDS (e.g. reviewed in [18]–[21]) and is therefore target of vaccination studies [22]. Nef enhances viral replication and accelerates the immune evasion of infected cells by manipulating transport as well as signaling pathways [23]–[27]. Nef-induced modulation of cellular signaling and transport pathways results in altered surface expression of cell surface receptors like CD4 and major histocompatibility complex class I and II or chemokine receptors [25], [28]–[32]. Also, interactions of Nef with components of the host cell cytoskeleton are documented that lead to rearrangements during e.g. the immunological synapse formation [33]. The majority of these Nef activities require a membrane localization of the protein that is supported by its N-myristoylation [34], [35].

We used the single vector system pETDuet-1 (Novagen) for cloning and coexpression of HIV-1 *nef* and human N-myristoyltransferase (*hNMT-1*) genes. For production of both proteins the pETDuet-1 recombinant was transferred to *E. coli* BL21-CodonPlus(DE3)-RIL competent cells (Stratagene) containing extra copies of the *argU*, *ileY* and *leuW* tRNA genes for improved translation efficiency of AT-rich heterologous proteins. Our strategy avoids the need for a time- and sometimes cost-intensive codon optimization of the gene of the myristoylation target without increasing the number of antibiotic resistance markers beyond two. High yield production and efficient purification of completely myristoylated Nef is demonstrated. The proposed strategy presents a general and highly efficient way for production of heterologous N-myristoylated proteins in *E. coli*.

## Results and Discussion

### High-yield production of N-myristoylated Nef

**Figure 1:**
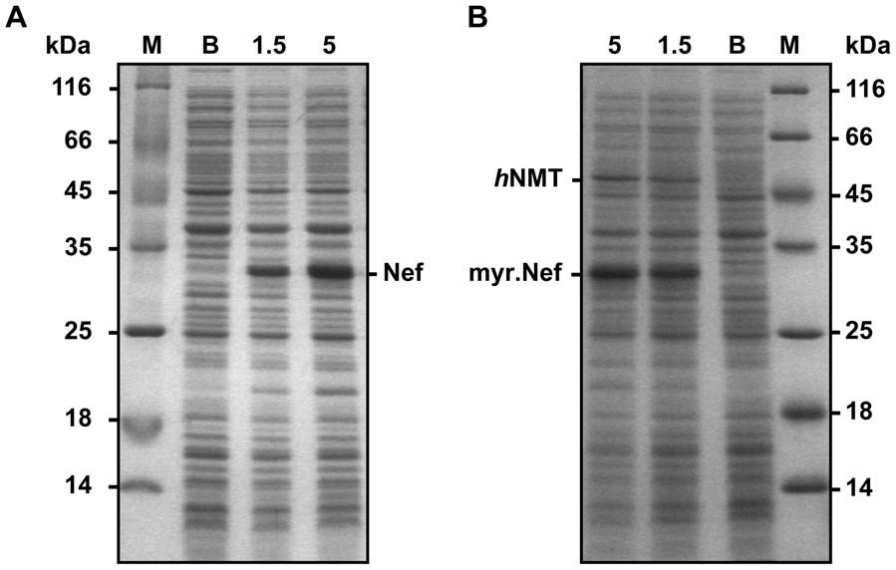
SDS-PAGE of cells expressing Nef (A) or myristoylated Nef (myr.Nef) resulting from coexpression of Nef and *h*NMT (B). Aliquots of the expression cultures were taken before as well as 1.5 and 5 hours after IPTG induction. The corresponding lanes are labeled with “B”, “1.5” and “5”, respectively. In addition, protein molecular weight marker (“M”) was applied.

Vectors pETDuet-1Δ6His_hNMT_Nef and pETDuet-1Δ6His_Nef were used for expressions of the *nef* gene with and without subsequent myristoylation of Nef, respectively. High-level production of Nef in BL21-CodonPlus(DE3)-RIL cells after induction with IPTG is demonstrated by SDS-PAGE traces in Figure 1. An additional, less intense but clearly visible band arises after induction of cells transformed with pETDuet-1Δ6His_hNMT_Nef (Figure 1 B). This additional band runs at a height corresponding to the molecular weight of 48.1 kDa of *h*NMT(81–496), indicating successful coexpression of the truncated N-myristoyltransferase. The molecular weight of Nef (24641 Da) and myristoylated Nef (24851 Da) differs by less than 1%, resulting in virtually indistinguishable running behavior in SDS-PAGE (see Figure 1). Proof of successful myrstoylation of Nef was obtained from ESI-MS analysis (see below).

Each of the two expression cassettes in the bicistronic vector pETDuet-1 contains its own T7*lac* transcription promoter and a ribosome binding site. However, there is only one transcription terminator site located downstream of MCS2. Assuming that genes 1 and 2 had been cloned into the MCS1 and MCS2 sites of pETDuet-1, respectively, transcription should yield two different mRNAs. A shorter one is initiated at the T7 promoter immediately preceding gene 2 and a longer one is initiated at the promoter upstream of gene 1. Both transcripts should terminate downstream of gene 2. Thus, it can be expected that translation of gene 1 will occur exclusively from the bicistronic mRNA while gene 2 will be translated from both the short and long transcripts [36]. The significantly higher yield of Nef in comparison to *h*NMT(81–496) observed in the pETDuet-1Δ6His_hNMT_Nef expression (Figure 1 B) is in perfect agreement with the expectations. Interestingly, bicistronic vectors containing two target genes under the control of a single promoter that precedes both genes frequently show strongly reduced expression of the gene located more distant from the promoter site in comparison with a two promoter vector [37].

Purification of both Nef and myristoylated Nef was accomplished with a straightforward and efficient protocol. The His-tagged Nef protein was isolated on a Ni-NTA affinity matrix followed by enzymatic removal of the His tag by overnight incubation with GST-PreScission protease during dialysis. The GST-tagged protease, cleaved His tag, and His-tagged Nef fusion were removed from the target protein Nef employing GSH and Ni-NTA affinity columns prior to the final polishing step on a size exclusion chromatography (SEC) column.

**Figure 2:**
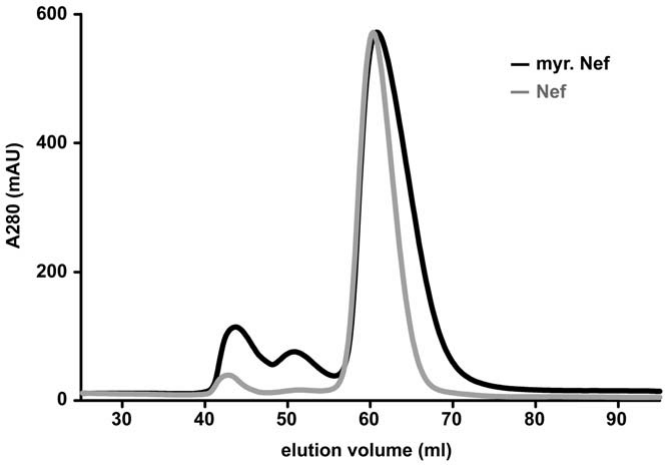
Gel filtration chromatograms documenting the final purification step of Nef and myristoylated Nef (myr. Nef). Protein samples were loaded onto a preparative HiLoad 16/60 Superdex 75 column, eluted with a flow rate of 1 ml/min and the absorbance at 280 nm was recorded. The elution profiles with maxima of the main peaks at around 61 ml indicate a monomeric state of Nef. Minor fractions eluting at around 44 and 51 ml reflect traces of aggregated protein and GST-PreScission protease, respectively.

**Figure 3:**
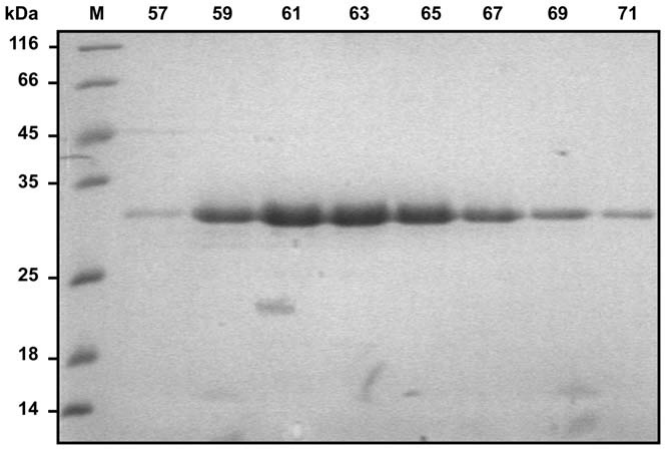
SDS-PAGE of 2 ml fractions collected during the final gel filtration of myristoylated Nef. Aliquots of the SEC fractions of myristoylated Nef (cf. Figure 2) were applied on a 15% SDS polyacrylamide gel followed by coomassie staining. SDS-PAGE revealed virtually pure Nef. Lanes are labeled with the elution volumes of the analyzed aliquots (57–71). Protein bands of the applied molecular weight marker (M) are labeled with the corresponding molecular weight in kDa.

An overlay of the SEC profiles documenting the final purification step of Nef and N-myristoylated Nef, respectively, is shown in Figure 2. The maxima of the main peaks elute at the same volume in both runs and the elution volume indicates monomeric Nef. The two small peaks eluting at 44 and 51 ml contain minor fractions of aggregated protein and GST-PreScission protease, respectively. SDS-PAGE analysis of the SEC fractions collected after applying myristoylated Nef to the column revealed virtually pure Nef in fractions 57 through 73 (Figure 3). The tail trailing of the main peak in case of myristoylated Nef obviously represents a more compact conformation in comparison with the major component. Breuer et al. proposed that the elution peak of myristoylated Nef reflects the coexistence of two molecular shapes - the acyl chain might be exposed in the “open” state, but inserted in a hydrophobic cavity in the “closed” or “compact” state [12]. Such a model was proposed earlier [38], and later it was shown that Nef indeed binds its own myristoylated amino-terminus [39]. In contrast, the elution peak of unmodified Nef is narrower and does likely reflect a single molecular shape. The observed behavior is reminiscent of the “myristoyl switch” mechanism - a conformational change that exposes or sequesters the myristoyl chain - which has been described for other myristoylated proteins [1], [2], [40], [41]. Very recently, a “myristoyl switch” mechanism driven by a combination of electrostatic attraction and membrane curvature sensing was proposed based on comprehensive Nef membrane binding data [42].

### Complete myristoylation of Nef

**Figure 4:**
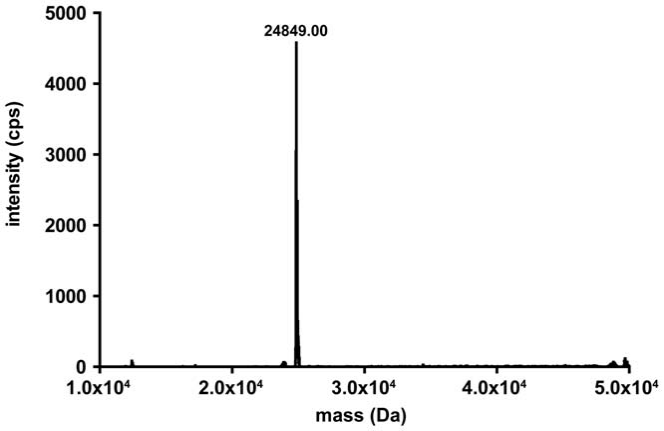
Section of mass spectrum of myristoylated Nef. Electrospray ionization mass spectrometry confirmed the successful myristoylation of Nef. Notably, no unmyristoylated Nef could be detected. The experimentally determined molecular weight of 24849 Da is in accordance with the theoretical value of 24851 Da.

High purity of the obtained myristoylated Nef was confirmed by ESI mass spectrometry. A single component with a molecular weight of 24849 Da was detected, which is in agreement with the theoretical value (24851 Da). Figure 4 shows a section of the mass spectrum. There were no indications on the presence of any unmyristoylated Nef (calculated molecular weight 24641 Da) in the mass spectrometry data, supporting that myristoylation was virtually complete.

The NH_2_-terminal amino acid sequence of Nef matches the consensus sequence of NMT protein substrates: Met-Gly-X-X-X-Ser/Thr-Lys/Arg-. The initiating Met is removed cotranslationally by methionine amino peptidase and Gly becomes the NH_2_-terminal amino acid. In general, N-myristoylation of proteins in mammalian cells occurs cotranslationally on the ribosome. Notably, NH2-terminally truncated *h*NMT lacking the ribosome targeting sequence retains *in vitro* catalytic activity [43]. Additionally it is known that the use of full length *h*NMT occasionally can result in rather incomplete myristoylation of the substrate [16].

### General applicability of the approach

Although coexpression of substrate and enzyme by a bicistronic vector has been shown before [44], [45] this is, to the best of our knowledge, the first report on efficient production of a myristoylated protein in *E. coli* utilizing a bicistronic vector. The pETDuet-1 vector was originally designed for production and analysis of protein-protein complexes but also allows co/post-translational modification of heterologous proteins. The *nef* gene was intentionally cloned into MCS2 while the gene encoding the enzyme was inserted into MCS1. As expected, this resulted in a significantly higher yield of Nef in comparison to the enzyme (cf. Figure 1 B). Importantly, the amount of active *h*NMT was sufficient for complete myristoylation of Nef. Our simple and efficient protocol resulted in ∼20 mg purified N-myristoylated Nef per liter of expression culture. This will allow continuation of the structural characterization of Nef complexes with other proteins [46]–[51] in a native like membrane environment.

Breuer et al. employed the more common dual vector strategy in combination with a codon optimized *nef* gene for high-yield production (∼30 mg protein per liter) of N-myristoylated Nef [12]. However, this increased yield required codon optimization of the gene coding for the protein of interest whereas our novel approach avoids this additional effort without increasing the overall number of required antibiotics. Moreover, only one instead of two transformation steps is needed.

The major advantage of our strategy is that only one antibiotic is required for stable maintenance of the single vector coding for both the enzyme NMT and the substrate Nef. The second antibiotic employed in our protocol allows convenient protein expression in cell lines that contain an extra plasmid for production of rare tRNAs. However, a second antibiotic is not required for expression of the dual vector in standard BL21 competent cells. In fortunate cases, the two original target genes will express at high levels in standard BL21 competent cells. Alternatively, codon-optimized genes may be utilized to boost expression in such cells without the need for a second antibiotic. In contrast, the dual vector strategy with separate plasmids for substrate and enzyme always requires a minimum of two different antibiotics. Further, use of the bicistronic vector with two transcription promoter and ribosome binding sites but only one transcription terminator site allowed us to channel the bacterial resources mainly towards production of the target protein Nef while only a small but sufficient amount of enzyme was produced. Finally, the use of a single instead of two plasmids for expression of substrate and enzyme eliminates the potential problem of incompatible ORIs that may arise in two plasmid systems.

In summary, the single vector based strategy demonstrated here for the expression of N-myristoylated HIV-1 Nef in *E. coli* offers a simple, general, easy to use, straightforward and efficient alternative to the previously described dual plasmid systems.

## Materials and Methods

### Vector preparation and cloning

The dual gene expression vector pETDuet-1 (Novagen) carries the pBR322-derived ColE1 replicon, the *lac*I gene, the ampicillin resistance gene, and two multiple cloning sites MCS1 and MCS2. Each of the two MCS is preceded by a T7*lac* promoter and a ribosome binding sequence. First, we removed the sequence coding for the NH2-terminal hexahistidine (His) tag of the gene product in the MCS1 cassette by cleavage of the original pETDuet-1 plasmid with restriction endonucleases *Nco*I and *Eco*RI, followed by blunt-end ligation. The resulting vector is referred to as pETDuet-1Δ6His in what follows.

The *nef* gene of HIV-1 isolate SF2 (SwissProt accession no. P03407) was PCR-amplified from the previously described vector pUbi-Nef_2–210_ [39] using primers containing the *Nde*I and *Mun*I restriction sites at the 5′ and 3′ ends, respectively. The 3′ primer represents the reverse complement of the nucleotide sequence coding for the last seven COOH-terminal amino acid residues of Nef, the PreScission protease cleavage site (LEVLFQGP), a His affinity tag and a stop-codon followed by the *Mun*I site. The amplified *nef* gene was cloned into MCS2 of the modified pETDuet-1 vector utilizing the *Nde*I and *Mun*I sites. The obtained vector pETDuet-1Δ6His_Nef was employed for production of nonmyristoylated Nef.

A gene coding for residues 81 to 496 of human N-myristoyltransferase (*hNMT-1*) was PCR-amplified from the vector pBB218 [52] and cloned into MCS1 of the pETDuet-1Δ6His_Nef vector between the *Eco*RI and *Hind*III restriction sites. NH_2_-terminally truncated hNMT(81–496) is catalytically active but lacks the ribosome binding site of the full length protein [43]. The resulting vector pETDuet-1Δ6His_hNMT_Nef was used for production of N-myristoylated Nef. All enzymes required for cloning were obtained from Fermentas (St. Leon-Rot, Germany).

### Protein expression and purification

E. coli strain BL21-CodonPlus(DE3)-RIL (Stratagene) was transformed with plasmid pETDuet-1Δ6His_Nef or pETDuet-1Δ6His_hNMT_Nef. Recombinant protein production was performed in LB medium supplemented with ampicillin (100 µg/ml) and chloramphenicol (34 µg/ml). Each 1 l expression medium was inoculated with an aliquot of a 50 ml overnight culture to an optical density of ∼0.1 at 600 nm (OD_600_). After the culture had reached an OD_600_ of 0.6 the temperature was reduced from 37°C to 28°C. Gene expression was induced at an OD_600_ of 0.8 by addition of a 1 M isopropyl-β-D-thiogalactopyranosid (IPTG) stock solution to a final concentration of 0.5 mM. For production of N-myristoylated Nef the *h*NMT substrate myristic acid (Sigma, Seelze, Germany) was added 10 min before induction to a final concentration of 50 µM. Myristic acid was supplied as a freshly prepared 5 mM stock solution containing 0.6 mM BSA (Sigma). The pH of the stock solution was adjusted with NaOH to 9 and briefly heated to 50°C for complete solubilization of myristic acid. Cells were incubated under gentle agitation (150 rpm) and harvested 5 hours after induction by centrifugation (5000 × g, 4°C, 30 min). Cell pellets were washed in 1× PBS buffer, spun down and stored at -20°C. Protein expression was checked by SDS-PAGE.

For protein purification cell pellets harvested from 1 l culture volume were thawn and resuspended on ice in 25 ml lysis buffer (20 mM Tris-HCl pH 8.0, 500 mM NaCl, 20 mM imidazole pH 8.0, 15 mM β-mercaptoethanol) supplemented with lysozyme (200 µg/ml) and protease inhibitor cocktail (Complete, EDTA-free; Roche). Cells were disrupted by sonification on ice using a Branson 250 sonifier equipped with a microtip (3 cycles of 30 s with 1.5 min of cooling on ice in between). The crude lysate was clarified by centrifugation (50000 × g, 4°C, 30 min). Subsequent purification steps were performed at 6°C ambient temperature. The supernatant was applied onto a gravity flow column (column volume (CV) of 5 ml) packed with nickel-nitrilotriacetic acid (Ni-NTA) agarose (Qiagen; Hilden, Germany) and pre-equilibrated with lysis buffer. Unbound material was removed by washing with 10 CV of lysis buffer followed by 10 CV of lysis buffer containing 30 mM imidazole. Nef or myristoylated Nef protein, respectively, bound via the His tag, was eluted by increasing the imidazole concentration in the buffer. Nef containing fractions were pooled and dialyzed against 20 mM Tris-HCl pH 8.0, 500 mM NaCl, 15 mM β-mercaptoethanol. The His tag was enzymatically removed with glutathion-S-transferase (GST)-tagged PreScission protease (GE Healthcare, Freiburg) during dialysis. Cleavage/dialysis was performed overnight utilizing ∼1 mg of protease per 5 mg of Nef. Separation of the His tag, any uncleaved His-tagged Nef fusion protein and the GST-tagged PreScission protease from untagged Nef protein was accomplished with two gravity columns (CV of 5 ml each) in series. The first column was packed with GSH-Sepharose 4 B (GE Healthcare) and the second one with Ni-NTA agarose. Nef containing flow-through fractions were combined and applied to a HiLoad 16/60 Superdex 75 prep grade column (GE Healthcare) pre-equilibrated with 20 mM Tris-HCl pH 8.0, 150 mM NaCl, 15 mM β-mercaptoethanol. The gel filtration column was operated on an ÄKTA purifier system at 10°C with a flow rate of 1 ml/min. Protein sizes were estimated based on a column calibration with a mixture of low molecular weight proteins (gel filtration calibration kit LMW; GE Healthcare). Nef containing fractions were pooled and concentrated with centriprep devices (Millipore). Cleavage, separation efficiency and final purity were evaluated by SDS-PAGE analysis. Nef solution was kept at 4°C for short-term storage or shock frozen in liquid nitrogen prior to long-term storage at −80°C.

### Electrospray ionization (ESI) mass spectrometry

Purity and myristoylation efficiency of the acylated Nef was checked by ESI mass spectrometry. Prior to analysis with an ESI-QqTOF instrument (QSTAR XL; Applied Biosystems, Darmstadt) low molecular weight buffer components were removed on a reverse phase column (Resource RPC 1 ml; GE Healthcare) operated with a linear gradient from 100% buffer A (100% ddH_2_O, 0.1% (v/v) TFA) to 100% buffer B (80% acetonitrile, 20% ddH_2_O, 0.08% (v/v) TFA) over 20 column volumes. MS analysis was performed at the BMFZ core facility of the University of Düsseldorf.
